# Life Cycle Analysis of Coaxial Layered Fiber Spinning for Wind Turbine Blade Recycling

**DOI:** 10.1021/acssusresmgt.4c00434

**Published:** 2025-04-25

**Authors:** M. Taylor Sobczak, Gengyang Li, Arunachalam Ramanathan, Sri Vaishnavi Thummalapalli, Varunkumar Thippanna, Lindsay B. Chambers, Taylor Theobald, Hongyue Sun, Stephen Nolet, Ke Li, Kenan Song

**Affiliations:** † Mechanical Engineering, College of Engineering, 1355 University of Georgia, Athens, Georgia 30605, United States; ‡ Environmental Engineering, College of Engineering, University of Georgia, Athens, Georgia 30605, United States; § TPI Composites, Scottsdale, Arizona 85253, United States

**Keywords:** LCA, wind turbine, fiber spinning, composite fiber, recycling

## Abstract

This article explores the environmental sustainability of recycling decommissioned wind turbine blades to produce polyacrylonitrile fiber. By comparing greenhouse gas emissions across various scales of production in different regions, including the US and Europe, the study highlights how cleaner energy grids, such as those in France, can substantially reduce the carbon footprint. The carbonization and graphitization stages, identified as highly energy-intensive, underscore the need for energy-efficient techniques and alternative energy sources. The study reveals significant reductions in greenhouse gas emissions with scalable production, demonstrating US production emissions reduced to 3.89 kg CO_2_ equiv/kg fiber and European production to 3.28 kg CO_2_ equiv/kg fiber from a lab scale of at least one order of magnitude higher. The findings emphasize the importance of sustainable raw materials, green chemistry, and renewable energy in enhancing the sustainability of carbon fiber production and promoting a circular economy in wind energy.

## Graphical abstract

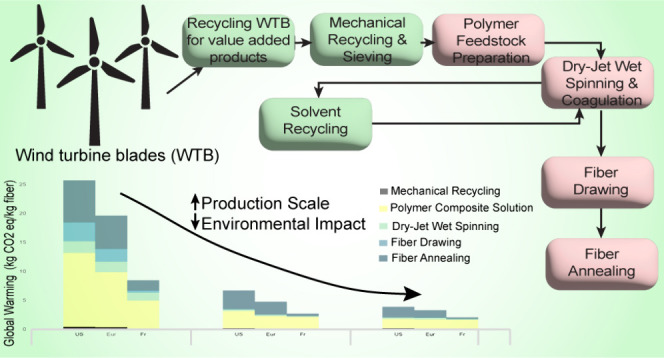

## 1 Introduction

In the pursuit of sustainable energy solutions, wind power has emerged as a prominent source of renewable energy, contributing significantly to global efforts to mitigate climate change and reduce dependence on fossil fuels.^1^ Central to the wind energy sector is the production of WTBs (wind turbine blades), which play a pivotal role in harnessing wind energy and converting it into electrical power.^2^ Global wind energy total installed capacity has increased from 25 GW to 651 GW from 2001 to 2019,^3^ and thus total cumulative WTB waste will grow from 789,000 tons in 2021 to 43 million tons by 2050.^4^ Despite their pivotal role in advancing sustainable energy production, wind turbine blades eventually reach the end of their operational lifespan, presenting the industry with a pressing and multifaceted challenge – the effective recycling and disposal of these massive composite structures.^5^ Wind turbine technology has become increasingly attractive as advancements have enabled clean and decentralized energy production for remote areas.^6^ With the proliferation of wind farms globally, the volume of decommissioned wind turbine blades is on the rise, necessitating urgent attention and innovative solutions to address their end-of-life management.^7^

The disposal of decommissioned wind turbine blades poses significant environmental, economic, and logistical challenges.^8^ Traditional disposal methods, such as landfilling and incineration, raise concerns about resource depletion, environmental pollution, and waste management.^9^ The global volume of waste from decommissioned wind turbine blades is expected to reach 200,000 tons annually, with most of this waste projected to end up in landfills.^10^ Landfilling, while once a common practice, consumes valuable space and poses risks to soil and groundwater contamination due to secondary contamination issues including leachate contamination, microplastic/fiber release, and waste cross contamination to name a few.^11–13^ Similarly, incineration, while capable of reducing the volume of blades, presents environmental risks associated with air pollution and emissions of toxic pollutants.^14^ As the lifespan of wind turbine blades reaches its end, the industry faces a pressing challenge of effectively recycling and managing the disposal of these large, composite structures. Conventional disposal methods pose substantial environmental and economic concerns, necessitating the exploration of alternative recycling techniques.^15,16^ This calls for the need to find a sustainable pathway to dispose of this complex composite waste, which could include techniques such as upcycling to create new products from waste, repurposing, and waste to energy (WTE) techniques such as pyrolysis and gasification.^17,18^ Pyrolysis and other WTE other the ability to utilize a wide variety of waste plastics to produce syngas, biochars, and bio oils which are useful products for energy and reagent production.^19^ Pyrolysis and reuse of glass fiber reinforced polymer (GFRP) has been studied,^20^ but these solutions only present pathways for recovering the matrix or generating energy from the resin, while the glass fibers themselves often suffer from degraded mechanical properties, limiting their direct reuse in high performance applications.

Fiber spinning and textile engineering can facilitate the recycling of waste materials by converting them into high-value fibers and textiles.^21^ Fiber engineering techniques, such as electro, melt, and wet spinning have all demonstrated the ability to create high value fibers with natural or recycled feedstocks for advanced applications.^22,23^ These processes not only help in minimizing waste but also promote the development of high performance sustainable materials, reducing the environmental footprint of various industries.^24^ For instance, waste materials can be processed into fibers for use in clothing, construction, and automotive applications, providing a versatile solution to the growing problem of waste management. As compared to these traditional disposal methods, our group’s previous research demonstrated a promising alternative for wind turbine blade recycling through a coaxial layered fiber spinning process.^25^ This innovative technique involves the mechanical disassembly of decommissioned blades, followed by the separation and recovery of their constituent materials through mechanical recycling and spinning technology. By leveraging coaxial spinning, it becomes possible to utilize materials such as mechanically recycled fiberglass from WTBs with low aspect ratios and defect ridden resin inclusions, to manufacture new high performance composite products. Additionally, incorporating glass fiber wastes into the polyacrylonitrile (PAN) polymer matrix enhances the mechanical and thermal properties, potentially lowering the stabilization and carbonization temperatures, which can result in a lower carbon footprint and more cost-effective manufacturing.

PAN fibers are the most widely used precursors for carbon fiber (CF) production, accounting for more than 90% of global CF production.^26,27^ The predominant preference for PAN as a precursor is largely attributed to its facile processing conditions, rapid processing speed, high carbon yield, and superior tensile strength.^28^ Additionally, PAN-based carbon fibers exhibit excellent thermal stability and resistance to chemical and environmental degradation, making them ideal for high-performance applications.^29^ The versatility of PAN fibers also allows for modifications to their molecular structure, enabling the fine-tuning of fiber properties to meet specific requirements in the aerospace, automotive, and sports equipment industries.^30^

These attributes are precisely why we have chosen to target a PAN matrix for our coaxial composite fibers in our previous work, as they offer an optimal balance of performance, processability, and industry maturity, facilitating ease of commercial implementation. Our group successfully utilized WTB waste products destined for landfills to produce high-value fibers with superb mechanical properties and achieved a 24.4% increase in strength and a 17.7% increase in modulus compared to virgin PAN fibers.^25^

In this context, the application of life cycle assessment (LCA) offers a systematic framework to evaluate the environmental sustainability of our novel coaxial layered fiber spinning^25^ compared to traditional disposal methods. By quantifying environmental impacts across the recycling process—from blade disassembly to material recovery and reprocessing—LCA provides valuable insights to inform decision-making and drive the adoption of environmentally responsible practices in wind turbine blade end-of-life management. This study aims to investigate the environmental performance of coaxial layered fiber spinning for wind turbine blade recycling, with a focus on mitigating environmental impacts. Through rigorous analysis of environmental metrics, including carbon footprint, energy consumption, and resource utilization, the study seeks to highlight the potential of this recycling approach to promote sustainability in wind energy. Quantified data will be employed to underscore the environmental benefits and viability of coaxial layered fiber spinning, thereby advancing the discourse on sustainable strategies for wind turbine blade recycling and circular economy integration.

Few studies have analyzed and quantified the environmental impact of novel, noncommercial techniques for creating value-added products from waste materials, such as our innovative fiber spinning method. By addressing this gap, our work contributes to a deeper understanding of the sustainability potential of these approaches. This research not only highlights the feasibility of waste valorization but also provides critical insights into the life-cycle impacts of emerging technologies. Such findings are essential to guide future developments and promote environmentally responsible innovation. This study aims to

1. Examine the environmental impact of our process in terms of TRACI categories.
2. Quantify the impacts of each process.
3. Assess the relationship between variables to determine future research targets.

## 2 Methods

### 2.1 Life Cycle Assessment Methodology

LCA is an environmental impact assessment method that considers the material and energy interactions over the life cycle of a product or process that impacts the environment. The LCA methodology used in this analysis follows international guidelines set by the International Organization for Standardization (ISO) in ISO 14040–14044. These guidelines set the framework for LCA and consist of four major components: goal and scope definition, life cycle inventory (LCI), life cycle impact assessment (LCIA), and interpretation of results. The LCA approach was taken in this analysis at the end-of-life to the gate. The final output is polyacrylonitrile and glass fiber coaxial composite fibers (PAN/GFs). According to the ISO 14040–14044 framework, the procedure for the analysis is as follows:

1. Define the system boundary of our manufacturing process.
2. Conduct inventory analysis and impact analysis.
3. Apply the upscaling technique for larger production scales.
4. Analyze energy consumption and resource utilization of the coaxial layered fiberspinning process to obtain PAN/GFs at different production scales.

### 2.2 Functional Unit & System Boundaries

The functional unit is the production of 1 kg of carbon composite fibers using wind turbine blade waste as fillers (1 wt %). The system boundary follows our novel coaxial fiber spinning process outlined in Figure 1.^25^ The fiber spinning, annealing, and heat treatment procedures begin with preparing a PAN solution by dissolving the PAN copolymer in Dimethylformamide (DMF) and stirring it at 130 °C until a transparent solution is obtained. This solution is deaerated, transferred into a metal syringe, and injected into an in-house customized spinneret for fiber formation. For three-layered fibers, a unique coaxial spinneret is used, with the interior and exterior layers containing PAN solution and the middle layer containing varying concentrations of GF fillers dispersed in the PAN solution. The spinning process involves injecting the solution into an air gap before entering a methanol coagulation bath, facilitating fiber extension and molecular alignment. The as-spun fibers undergo hot drawing through baths of water and silicone oil at controlled temperatures to maximize molecular extension and remove solvents. This process aligns the macromolecules parallel to the fiber axis and collapses voids, resulting in defect-free fibers. The drawn fibers are then heat-treated in a tube furnace in an oxidative atmosphere at 250–400 °C to eliminate residual stresses and stabilize the fibers, enhancing their quality and performance.

**Figure 1:**
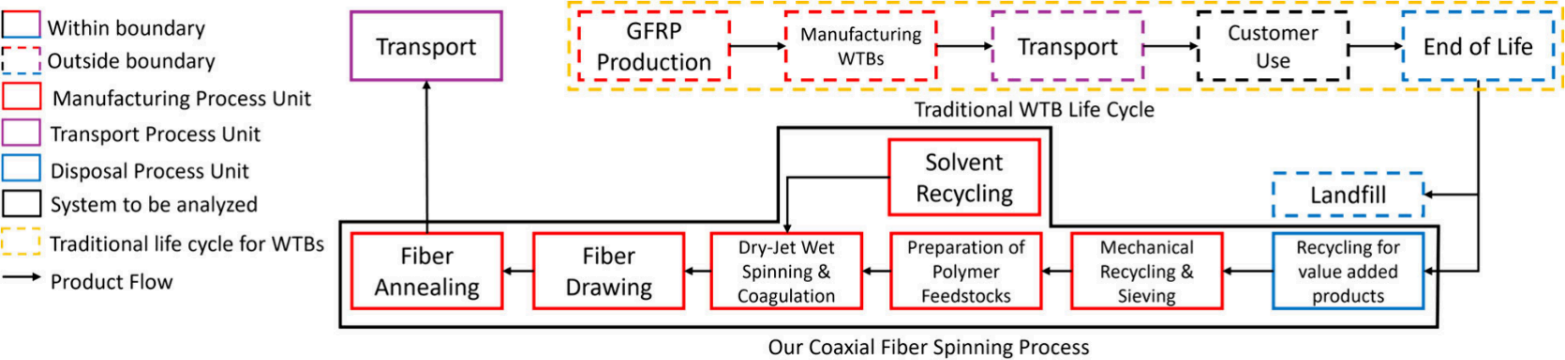
System boundary for coaxial PAN/GF fiber production from glass fiber reinforced plastics (GFRPs) from WTBs.

The production flow is as follows: transport of wind turbine blade waste (glass fiber reinforced plastic) to the precursor production plant, mechanical recycling, preparation of polymer feedstocks, which includes mixing and heating of solution, dry-jet wet spinning and coagulation, fiber drawing, fiber annealing, and final transport. Heating is required in all processes except for mechanical recycling. The heating process parameter conditions are based on our production method, as described in our previous publication. Here is a brief overview of the fiber production method. Stabilization temperature can vary from 250–400 °C, but for consistency, we have chosen to model it at 300 °C.

A system expansion approach was used to credit the recycled material. For instance, credits for diverting glass fiber composites from landfills and producing high-modulus PAN fibers were applied using a “1-to-1 avoided product” assumption (as discussed in Section 3.1). This ensures that the recycling process’s environmental benefits are fully integrated into the system boundary without requiring arbitrary allocation rules, which is discouraged by the ISO standard. The system boundary includes all stages from the collection of decommissioned wind turbine blades through to the production of recycled PAN fibers. This boundary ensures that all recycling-related inputs (e.g., energy, transportation) and outputs (e.g., recovered materials) are accounted for.

### 2.3 Inventory

Inventory analysis was performed to assess the carbon footprint, energy consumption, and resource utilization during the fiber-spinning process of wind turbine blades. The main inventory uncertainty was power scaling. From similar literature on CF precursor production, a simple power scaling formula was presented, *P* = *cS*_*x*_^*f*^, where *f* and *c* vary for different equipment types, *P* represents the power, and *S*_*x*_ represents the target scale.^31^ In our analysis, we used different values for *f* and *c*. For heating and stirring equipment *f* and *c* were 0.5688 and 0.6045 respectively, and for drying equipment, they were 0.3222 and 0.6858 as obtained from Kawajiri and Sakamoto.^31^ Our lab scale was able to generate about 100 g per production run, and we chose to analyze a mid-scale production of 100 kg and a full-scale production of 1000 kg. Carbon fiber production facilities are capable of producing hundreds of thousands of metric tons per year, encompassing various types such as PAN-based, pitch-based, high modulus (HM), high strength (HS), and intermediate modulus (IM). Given that our PAN precursors have demonstrated high modulus, we assumed that HM PAN fibers represent a smaller subset of a facility’s total production, thus deducing batch sizes of 1000 kg, with production times estimated at 2–10 h for this quantity in a full-scale facility.

All inventory data was obtained from our lab scale data for the fiber production stages Table 1.^25^ Full scale calculated inventory can be found in Table S1. Transportation estimates were calculated based on basic fleet freight to and from primary wind farm locations, recycling facilities, and CF precursor producer plants. Process gas emission-based data was obtained from Sakamoto et al.^32^ The material, chemical, and energy data during the recycling process as delineated as solid line in the system diagram are all primary data from our lab. The upstream and downstream processes were secondary data. Most secondary data for this study was sourced from the SimaPro. Secondary data for this study was sourced from the SimaPro software using the ecoinvent database. This database provides comprehensive life cycle inventory data, including emissions, resource usage, and environmental impacts for various materials and processes. The use of ecoinvent ensures consistency and reliability for upstream and downstream processes that were not directly measured in the study.

**Table 1:** Life Cycle Inventory Data for Our Novel Coaxial Fiber Spinning Process^25^ ^,^ ^a^

Task	Amount Produced	Material Requirements	Unit	Quantity
Mechanical	1 kg	GFRP from WTBs	kg	1.1
Recycling		Transport of wind turbine blades	km	1700
		Electricity	MJ	3.4
Preparation of	1 kg	PAN polymer	kg	0.773
Polymer		DMF Solvent	kg	0.322
Feedstock		Electricity	MJ	43.8
Dry Jet Wet	1 kg	Methanol	kg	0.083
Spinning		Electricity	MJ	7.7
		Methanol off gas	kg	0.083
Fiber Drawing	1 kg	Silicone Oil	kg	0.064
		Electricity	MJ	11.2
Fiber	1 kg	Transport to consumer	km	200
Annealing		Electricity	MJ	115.2
		Off gas production (HCN, CO, CH_4_, and hydrocarbons)	g	70
Hazardous Waste Disposal for all processes	N/A	Hazardous waste	kg	1.25

a All data is for lab-scale production directly based on our lab-scale production data.

Solvent recapture and recycling systems used at production-level scales are capable of achieving 95% solvent reusability.^33^ This high rate of solvent recovery significantly reduces both environmental impact and operational costs, making the process more sustainable and economically viable. Implementing such systems not only enhances the efficiency of fiber production but also aligns with our goal to reduce the overall environmental impact of PAN fiber production. The solvent used for the spinning process contains around 10% DMF and 90% methanol.^25^ The methanol from the solvent could be distilled and recovered with heat energy.^34^

Although transportation was included in our analysis, it plays a minimal role in the overall environmental impact, as waste product represents a small fraction of the overall functional unit. The raw materials are transported from Texas/Oklahoma to the Toray facility in Alabama, covering a distance of approximately 1700 kilometers. Fleet average diesel-powered trucks are used to transport semi-cut wind turbine blades to the waste recycling facility, and then to the precursor facility. The region between Texas and Oklahoma was chosen because there is a large number of wind farms in this area^35^ and it is close to major CF manufacturers.

### 2.4 Life Cycle Impact Assessment

Life Cycle Impact Assessment (LCIA) is a qualitative and quantitative assessment of the environmental impact of a process based on resource, energy consumption data, and various emission data. In this study, SimaPro 7.0 software was used to build the LCA model and perform the environmental impact assessment using the EPA’s Tool for Reduction and Assessment of Chemicals and Other Environmental Impacts (TRACI) methodology. A total of six environmental and 3 human impact categories were considered in this study. The environmental impact categories include ozone depletion (OD, CFC-11 equiv), global warming potential (GWP, kg CO_2_ equiv), acidification (AD, mol H+ equiv), marine eutrophication (ME, kg N equiv), smog formation (SF, kg O_3_ equiv), and ecotoxicity (ET, CTUe). The human impact categories include carcinogens (C, CTUh), noncarcinogens (NC, CTUh), and respiratory effects (RE, kg PM10 equiv).

## 3 Results

### 3.1 Overall Impact

The overall impact of all processes was calculated by the EPA’s TRACI 2.1 methodology. TRACI was chosen as it contained all of the characterizations of environmental stressors we were interested in, including global warming potential and ozone depletion.^36,37^ Tables 2 and 3 represent the total overall impacts of each of the processes for both the US and European energy grids. All values of Tables 2 and 3 are calculated based on the production of 1 kg of fiber.

**Table 2:** Summary of the Environmental Impacts for the Production of 1 kg of PAN-GF Composite Fiber from Wasted Wind Turbine Blades Based on the US Energy Grid

Metric	Unit	Lab Scale	100 kg	1000 kg
Ozone depletion	kg CFC-11 equiv	1.28 × 10^–6^	3.43 × 10^–7^	1.22 × 10^–7^
Global warming	kg CO_2_ equiv	25.7	6.09	3.29
Smog	kg O_3_ equiv	1.63	0.244	0.038
Acidification	mol H^+^ equiv	9.08	1.68	0.546
Eutrophication	kg N equiv	0.1805	0.0202	0.0101
Carcinogenics	CTUh	1.30 × 10^–6^	2.58 × 10^–7^	1.31 × 10^–7^
Noncarcinogenics	CTUh	2.84 × 10^–6^	6.26 × 10^–7^	3.07 × 10^–7^
Respiratory effects	kg PM10 equiv	0.0244	0.0052	0.00189
Ecotoxicity	CTUe	12.3	4.43	2.21

**Table 3:** Table Summarizing the Environmental Impacts for the Production of 1 kg of Fiber Based on Europe’s Energy Grid

Metric	Unit	Lab Scale	100 kg	1000 kg
Ozone depletion	kg CFC-11 equiv	1.33 × 10^–6^	2.91 × 10^–7^	1.17 × 10^–7^
Global warming	kg CO_2_ equiv	18.9	4.16	2.68
Smog	kg O_3_ equiv	0.960	0.144	0.00687
Acidification	mol H^+^ equiv	3.80	0.699	0.130
Eutrophication	kg N equiv	0.144	0.0225	0.0131
Carcinogenics	CTUh	1.26 × 10^–6^	2.21 × 10^–7^	1.31 × 10^–7^
Noncarcinogenics	CTUh	2.64 × 10^–6^	5.14 × 10^–7^	2.95 × 10^–7^
Respiratory effects	kg PM10 equiv	0.0146	0.00306	0.00114
Ecotoxicity	CTUe	19.4	3.56	2.08

For full-scale US production of our coaxial composite, PAN precursor fibers had environmental impacts of 1.22 × 10^–7^ kg CFC-11 equiv ozone depletion, 3.29 kg CO_2_ equiv global warming potential, 0.038 kg O_3_ equiv smog, 0.546 mol H^+^ equiv acidification, 0.0101 kg N equiv eutrophication, 1.31 × 10^–7^ CTUh carcinogenics, 3.07 × 10^–7^ CTUh noncarcinogenics, 0.00189 kg PM10 equiv respiratory effects, and 2.21 CTUe ecotoxicity. For full-scale European production of the PAN fibers had environmental impacts of 1.17 × 10^–7^ CFC-11 equiv ozone depletion, 2.68 kg CO_2_ equiv global warming potential, 0.00687 kg O_3_ equiv smog, 0.130 mol H+ equiv acidification, 0.0131 kg N equiv eutrophication, 1.31 × 10^–7^ CTUh carcinogenics, 2.95 × 10^–7^ noncarcinogenics, 0.00114 kg PM10 equiv respiratory effects, and 2.08 CTUe ecotoxicity. Fiber produced with European energy mixes led to lower environmental impacts across all metrics.

All scenarios showed that as the scale increased the total impact in each category decreased. The scales were chosen to cover the basis of the lab-scale, mid-scale, and full-scale production lines. Across Tables 2 and 3 the trends were consistent between the two grids. It is interesting to note that the *ozone depletion, smog, and carcinogenic effects* were essentially net zero at full-scale production for both US and European operations. End-of-life recycling and final product credits were applied to all processes, as our in-house fiber spinning method was developed to recycle harsh wind turbine blade waste and create high-performance, value-added PAN precursor fibers. The recycling credit was calculated based on the amount of glass fiber composite waste diverted from landfills. Credits for the final product were determined using standard HM PAN precursors. These credits were incorporated into the SimaPro model as “avoided” products.

When production increases from the lab to the production scale, there is a steady decreasing trend. Ozone depletion decreases from 1.28 × 10^–6^ kg CFC-11 equiv to 1.22 × 10^–7^ kg CFC-11 equiv based on the US energy grid and from 1.33 × 10^–6^ kg CFC-11 equiv to 1.17× 10^–7^ kg CFC-11 equiv based on the European energy grid, showing roughly a 90%+ decrease. All environmental impact categories showed a significant decrease as scale increased, despite the production area. There was no significant difference *in ozone depletion, eutrophication, carcinogens, and noncarcinogens* between the US and Europe. This can be attributed to electricity production having the largest impacts on categories such as *acidification* and *global warming potential*.^38^ These findings underscore the importance of considering not only the production scale but also the underlying energy sources and production methods in assessing environmental impacts across different regions.

### 3.2 Process Contribution

Figures 2 and 3 show the overall relative and normalized process impact of lab-scale and full-scale production for the US. At the lab- and full-scale production, the polymer solution has the largest impact on all impact categories.

**Figure 2:**
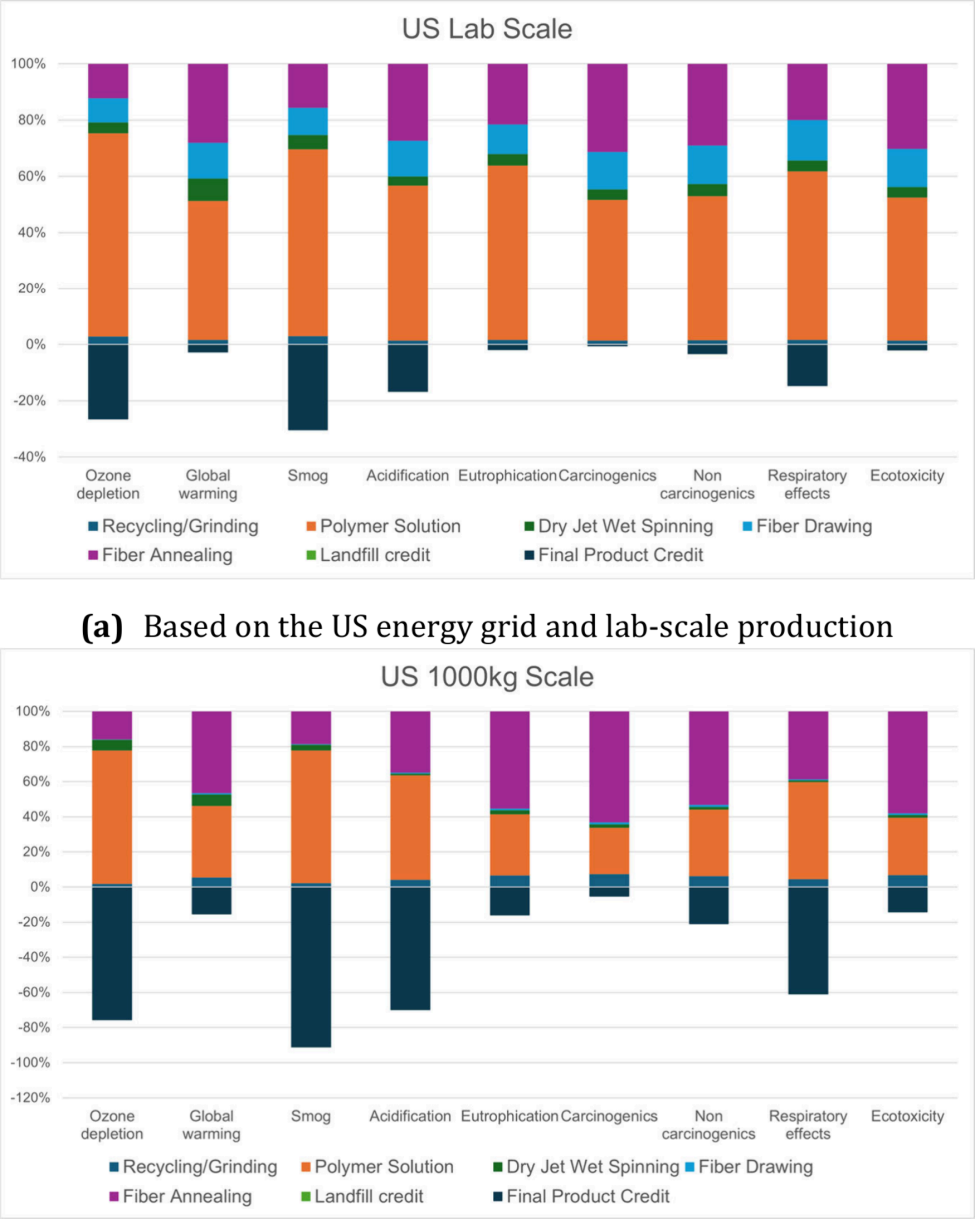
Relative process contributions normalized by total impact based on the lab- and full-scale production of US-based fibers.

**Figure 3:**
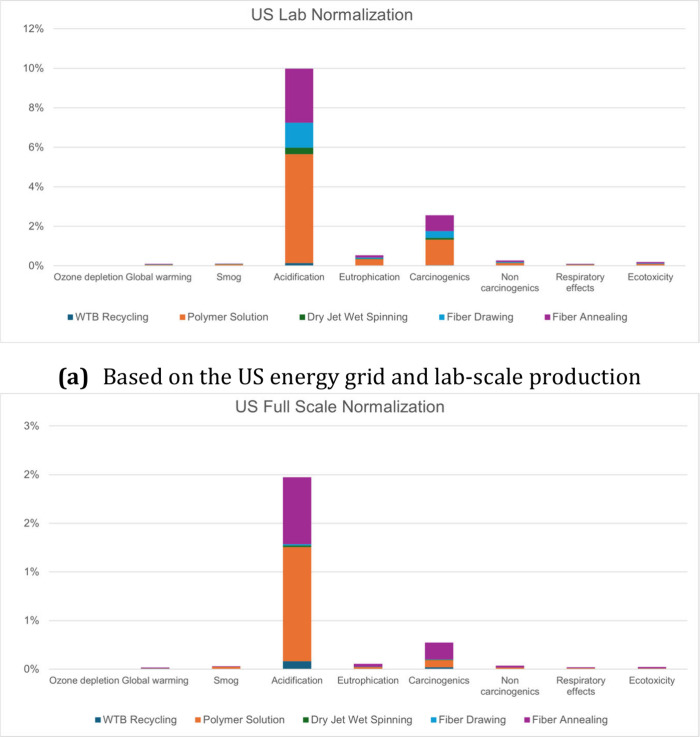
Process contributions normalized with normalization factors based on the lab- and full-scale production of US-based fibers. Normalization data is calculated based on the net results including impact and the recycling credits.

Looking into the lab-scale (Figure 2a) and full-scale (Figure 2b) relative contributions, there are some major differences between the two volumes. The polymer composite solution has the highest impact across almost every category for all impact categories across both scales. Fiber annealing accounts for a considerably larger impact across all categories, with full-scale production achieving around 20–50%. The impact of polymer solution also varied in US production, which could be attributed to the dirtier nature of the US energy grid, resulting in higher impacts on categories such as ozone depletion and acidification due to emissions of sulfur dioxide and nitrogen oxides from fossil fuel burning.^39^ Dry-jet wet spinning and fiber drawing had limited impacts on most impact categories for both production scales. While spinning processes require hazardous chemicals, such as methanol, we can recycle solvents at very high rates,^40^ limiting their environmental impact. The electricity inputs are also limited in these processes; for spinning, the extrusion of solutions requires limited energy, and for drawing, the only inputs are low-speed fiber winders and a small heated silicone oil bath. As the process scale increased, the kWh per kg of fiber produced drastically decreased, and raw materials and the highest energy-dependent properties began to take over the contributions.

Normalization comparison data (Figure 3) was based on the EPA’s 2008 TRACI 2.1 normalization factors.^41^ They are as follows: 0.16 kg CFC-11 equiv, 24,000 kg CO_2_ equiv, 1,400 kg O_3_ equiv, 91 mol H^+^ equiv, 22 kg N equiv, 0.0000505 CTUh for human toxicity cancer effects, 0.001037 CTUh for human toxicity non-cancer effects, 24 kg PM10 equiv, and 11,076.6 CTUe for ecosystem toxicity. All categories are based on the annual environmental impact of an average American. Based on normalization analysis, the main area that still needs to be focused on for reduction is acidification. The production of polymer solution and fiber annealing contribute significantly to the acidification potential for all production scales. Raw material procurement for PAN and DMF is quite environmentally harsh, and the synthesis processes, such as coal gasification, ammonia production, and others, produce SO_2_ and NOx as by-products. Heating and mixing of the polymer solution and the low-temperature stabilization processes are also highly energy-consumptive, and nonrenewable power production greatly impacts acidification potential.^42^ The process is heavily reliant on the use of solvents for processing. Traditionally produced solvents have harsh environmental impacts in areas such as acidification and carcinogenic effects.^43^ As process size increases, the overall normalization effects of acidification, eutrophication, and carcinogens decrease. Solvent recycling was not considered for the lab scale process, but was at the production scale process meaning less solvent was consumed and disposed of per kg fiber produced. The solvent recovery process was modelled with distillation, with input energy for recovery being natural gas (Table S2, Supporting Information). The value of heat energy was calculated via equation S1, Supporting Information. Calculated LCA data for recycling one unit of solvent is shown in Table S3, Supporting Information. Additionally, at larger production volumes, the process becomes inherently more energy efficient due to larger batch size, reduction in energy consumption can lead to the reduction of these metrics depending on the grid composition.^44^ Further reduction of environmental impact needs to focus on process efficiency and use of cleaner materials such as green polymers and solvents that can be produced from sustainable methods, have good recyclability, are biodegradable/biocompatible, and improved processing parameters.^45^

While the final PAN precursors have considerable global warming potential impact, their contribution to the average annual US capital impact is relatively insignificant. It is important not to underestimate the significance of this metric. By 2050, 2.2 million tons of cumulative WTB waste is expected to accumulate in the US,^46^ reinforcing the urgent need for sustainable disposal and recycling solutions. Landfill restrictions have already been put into action and as large volumes of wind turbine blade waste continue to amass it is critical to find a solution to divert waste in environmentally friendly ways.^47^ Our novel process offers a pathway to convert low-value waste scraps into high-value product CF precursors with improved mechanical properties.

### 3.3 GHG Emissions of Coaxial PAN Precursors

The greenhouse gas (GHG) emissions of coaxial precursor fiber preparation are shown in Figure 4. As shown in Figure 4, the US-based GHG emissions of the lab, 100 kg, and 1000 kg production scales are 25.68 kg CO_2_ equiv/kg fiber, 6.70 kg CO_2_ equiv/kg fiber, and 3.89 kg CO_2_ equiv/kg fiber, respectively. The synthesis of polymer solutions occupies the largest share of GHG emissions across all US processes with 57%, 51%, and 50% of CO_2_ emissions of all processes. This process consumes a considerable amount of electricity, as the solution is heated and mechanically stirred for 24 h. Additionally, this is the step of the process where polymers and solvents are acquired and consumed, requiring disposal of hazardous solvent wastes, although 95% solvent recyclability was used for both the DMF and methanol solvents.^33^ The production and synthesis of the PAN polymer and DMF also have a notable environmental impact, as the raw materials for these products are petrochemical-based.

**Figure 4:**
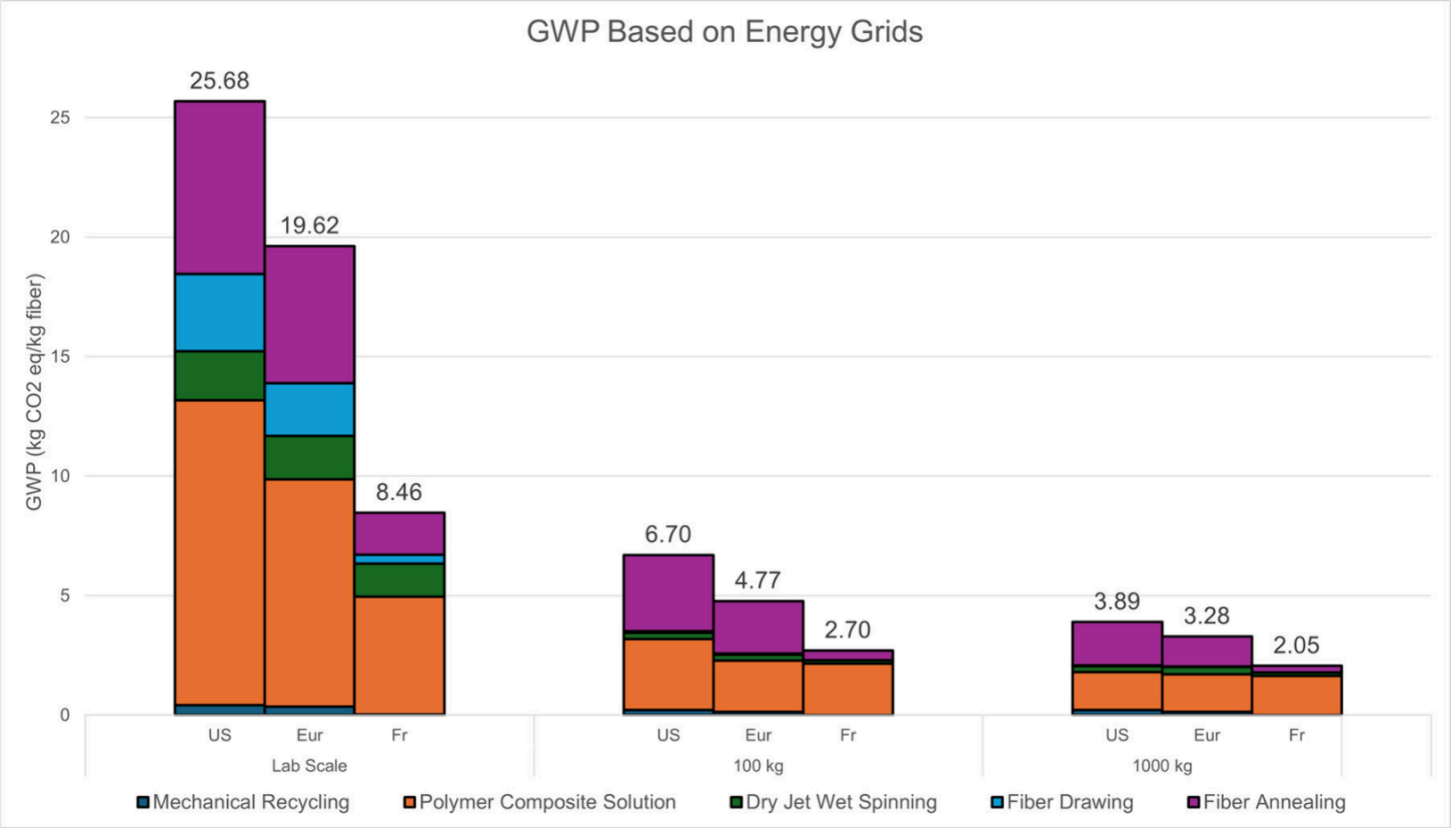
GHG emissions for coaxial PAN precursor fibers at different production scales. Process breakdown of GWP in kg CO_2_ equiv/kg fiber produced for US, Europe, and French power grids.

European production of fibers, Figure 4, saw a notable decrease in GHG emissions for both lab and production scales. The total emissions were 19.62 kg CO_2_ equiv/kg fiber, 4.77 kg CO_2_ equiv/kg fiber, and 3.28 kg CO_2_ equiv/kg fiber for lab, mid, and production scales, respectively. A 22.3%, 28.8%, and 15.6% decrease from US energy grids, respectively. This can be credited to Europe’s focus on renewable energy sources, thus having substantially cleaner power production.^48,49^ Studies have shown that the European Union has significantly invested in renewable energy infrastructure, resulting in a higher share of electricity generated from renewable sources compared to the US. In European production, polymer synthesis witnessed a similar trend, commanding the largest share across all production volumes. The three volumes accounted for 57.7%, 81%, and 86% of greenhouse gas emissions, emphasizing the processes’ substantial contribution to GHG emissions. This aligns with findings that polymer synthesis and processing are major contributors to the carbon footprint in fiber production.^50^

Transitioning to an ultraclean energy grid such as that of France further reduces the environmental footprint, as shown in Figure 4. GHG emissions at the lab to full production scale were 8.46 CO_2_ equiv/kg fiber, 2.70 CO_2_ equiv/kg fiber, and 2.05 CO_2_ equiv/kg fiber, respectively. France’s reliance on nuclear energy, which produces minimal GHG emissions, significantly lowers its carbon footprint compared to other countries.^51^ The impact of the polymer solution remains relatively the same across energy mixes, as raw material impacts are not affected by manufacturing location. The raw materials remain a limiting factor in the reduction of GHG emissions. Thus, while transitioning to cleaner energy grids can significantly reduce emissions during the production process, further research and development into sustainable raw materials are essential to achieving comprehensive environmental benefits.

The next highest process impact across all energy grids (Figure 4) was fiber annealing with 7.21 kg CO_2_ equiv/kg fiber at the lab scale and 1.81 kg CO_2_ equiv/kg fiber at the production scale, which accounts for 28.9 and 30.3% of total impacts, respectively. European production saw significant GHG emissions from fiber annealing as the annealing process is a major contributor to electricity consumption in the process, as fibers are heated in a low-temperature furnace for a couple of hours for stabilization purposes. This accounts for a substantial amount of electricity in our overall process.

Recycling for glass fiber production represents a negligible fraction at all scales across both grids (Figure 4). At US lab-scale and full-scale production, recycling only accounts for 0.411 kg CO_2_ equiv/kg fiber and 0.209 kg CO_2_ equiv/kg fiber, respectively, accounting for only approximately 1% of the total impact. This pattern is consistent for European power grids as well, with recycling accounting for 0.352 kg CO_2_ equiv/kg fiber and 0.143 kg CO_2_ equiv/kg fiber for lab and full-scale, respectively. Although other recycling methods can generate higher quality fibers,^52,53^ our group chose mechanical for its limited environmental impact compared to methods, such as chemical and thermal, thus limiting the overall GHG emissions of the process as the mechanical recycling process is negligible. While mechanical recycling does not yield high-quality raw materials, our novel manufacturing allows these low-value materials to be used in high-value products. Mechanical Recycling also has the highest technology readiness score of any current recycling technology,^54^ making it an excellent candidate for any location or market.

It can be noted in Figure 4. that the trend of GHG emissions decreases exponentially with scalability and the lower threshold is limited by other processes (polymer and solvent production). This phenomenon occurs because, while production efficiency improves with scale, the inherent environmental impact of raw materials remains constant per kg of fiber produced. Consequently, there is a minimum limit of environmental impact unless there are significant interventions in raw material production processes. Based on curve fitting and inventory analysis, the material level impact occurs around 3500 kg batches. This indicates that further scalability does not significantly reduce GHG emissions beyond a certain production volume due to the persistent impact of raw material sourcing.

Therefore, addressing the environmental impact of raw material production is crucial for achieving further reductions in GHG emissions. There has been ongoing research into green chemistry approaches for acrylonitrile production from biomass.^55,56^ Industrial implementation of these methods could further reduce the raw material GHG impact and lead to potential carbon-negative products. For example, by shifting to biomass-based acrylonitrile, not only can the reliance on fossil fuels be decreased, but the overall sustainability of the fiber production process can also be enhanced. Also, integrating renewable energy sources in production could amplify these benefits, contributing to a more sustainable and environmentally friendly manufacturing landscape.

As discussed previously the current main end-of-life process is disposal through landfilling or incineration. While landfilling has no benefit other than the disposal of the blade, incineration can be used for energy production. The main energy fuel source in the blades is the epoxy resin from the GFRP. The GWP for incineration of 1 kg of blade waste is 0.78 CO_2_ equiv, which is higher than a cleaner source such as lignin.^57^ Landfilling has also been shown to have a considerably higher impact at 0.22 CO_2_ equiv/kg blade waste across all impact markers as compared to other methods such as coprocessing.^58^ Although our process has a higher impact than conventional disposal methods, our group has successfully produced ultra-high-value-added products from landfill-destined waste, creating a sustainable pathway for CF precursor production and diverting potentially millions of tons of WTB from landfills.^59^

Across all regions, the polymer composite solution was the major contributor to GWP. As process size increases, the GWP impact of the polymer solution stabilizes, indicating that raw materials significantly affect the total process. Therefore, limiting the impact of raw materials is crucial for reducing the overall environmental impact. PAN was chosen for its high carbon yield and widespread industrial use.^60^ Additionally, exploring green polymers with higher carbon yields presents an opportunity to further reduce the environmental footprint. These sustainable alternatives, such as bio-based polymers, lignin, and cellulose-based materials,^61^ as well as those enhanced through methods like chemical modification and additive additions such as biochars, could provide higher carbon yields while decreasing reliance on traditional raw materials, leading to a more eco-friendly production process across all impact categories.

### 3.4 Impact of Higher-Temperature Carbonization and Graphitization

Carbonization and graphitization are critical steps in the production of carbon fibers from PAN precursor fibers. The carbonization process involves heating PAN precursor fibers in an inert atmosphere at temperatures typically ranging from 1000 °C to 1500 °C. This step removes non-carbon elements such as hydrogen, nitrogen, and oxygen, resulting in a carbon-rich structure. Carbonization significantly enhances the mechanical properties and thermal stability of the fibers, transforming them into strong and lightweight carbon fibers suitable for high-performance applications.^62^ Following carbonization, the graphitization process involves further heating the carbonized fibers to temperatures between 2000 °C and 3000 °C. Graphitization increases the degree of crystallinity and order within the carbon structure, producing a graphite-like arrangement. This process enhances the electrical and thermal conductivity of the carbon fibers and further improves their mechanical properties, making them ideal for use in aerospace, automotive, and other high-tech industries.^63^ The combined effects of carbonization and graphitization are crucial for optimizing the performance characteristics of carbon fibers. However, these processes are extremely energy-intensive, requiring significant thermal energy to achieve the necessary temperature and maintain the inert atmosphere. The high energy demands of carbonization and graphitization contribute to the overall environmental and economic costs of carbon fiber production, highlighting the importance of optimizing these heating steps to enhance the sustainability of the entire lifecycle.^64^

The carbonization and graphitization processes require holding material at different temperature zones for extended periods. These high-temperature processes require substantial electricity input, often reaching up to 340 MJ per kilogram of carbon fiber produced, and account for up to 33% of the total production cost of carbon fiber.^65^ The heating stages typically include an initial stabilization phase at around 200–300 °C, which was the stopping point of our initial fiber spinning and post-heat-treatment processes. The PAN fibers undergo cyclization reactions to become thermally stable. Following stabilization, the fibers are progressively heated to higher temperatures in the range of 1000–3000 °C in an inert atmosphere, ensuring that oxidation does not occur and graphitic layers form for high performance. During this phase, non-carbon elements such as hydrogen, nitrogen, and oxygen are removed, resulting in the formation of a highly crystalline, ordered graphite-like structure.

The carbonization process results in the emission of approximately 37.49 kg CO_2_ equiv for every kilogram of carbon fiber produced at lab scale, while at scale production of CFs had emissions of 19.85 CO_2_ equiv.^66^ This significant carbon footprint highlights the urgent need to develop more energy-efficient techniques and consider alternative energy sources to lessen the environmental and economic impact of the carbonization process. By enhancing the efficiency of the carbonization stage, we can greatly improve the sustainability of the entire carbon fiber life cycle. Research by Thippanna and others in the literature suggests that incorporating glass fibers, carbon nanotubes (CNTs), and other nanoparticles into the PAN matrix can significantly enhance the crystallization behavior of PAN fibers.^25,67^ These additives act as nucleating agents, promoting more orderly and extensive crystallization at lower temperatures.^68^ This improved crystallinity facilitates more efficient stabilization and carbonization processes,^69^ thereby reducing the energy requirements for subsequent heat treatment steps. For instance, the presence of glass fibers in the PAN matrix helps in the formation of more uniform and smaller crystalline regions, which can enhance the thermal stability of the fibers during the initial stages of heat treatment.^70,71^ Similarly, the addition of carbon nanotubes has been shown to increase the crystallization rate of PAN, leading to a higher degree of crystallinity before the onset of carbonization.^72,73^ This results in a more efficient transformation of PAN to carbon fibers, lowering the necessary temperatures and durations for both carbonization and graphitization steps, ultimately reducing the overall energy consumption of the process. By optimizing the crystalline structure of PAN through these additives, the entire production process of carbon fibers becomes more energy-efficient, making it not only more cost-effective but also more environmentally sustainable. These findings underline the potential of nanomaterials and other reinforcing agents in advancing carbon fiber manufacturing technology.

### 3.5 Global Warming Potential (GWP) Dependence on Locations

The choice of energy source significantly alters the Global Warming Potential (GWP) impact on any manufacturing process (Figure 5). For example, the U.S. energy mix primarily consists of nonrenewable sources, with coal, petroleum, and natural gas accounting for approximately 70%, while renewables contribute around 10–20%. In some regions, such as the Southeast, nuclear power comprises a significant portion of the energy mix, making up 24% of their total production,^74^ which can lead to cleaner production. In contrast, France generates the vast majority (more than 70%) of its power from nuclear energy,^75^ resulting in a much lower GWP compared to countries that rely more heavily on nonrenewable sources.

**Figure 5:**
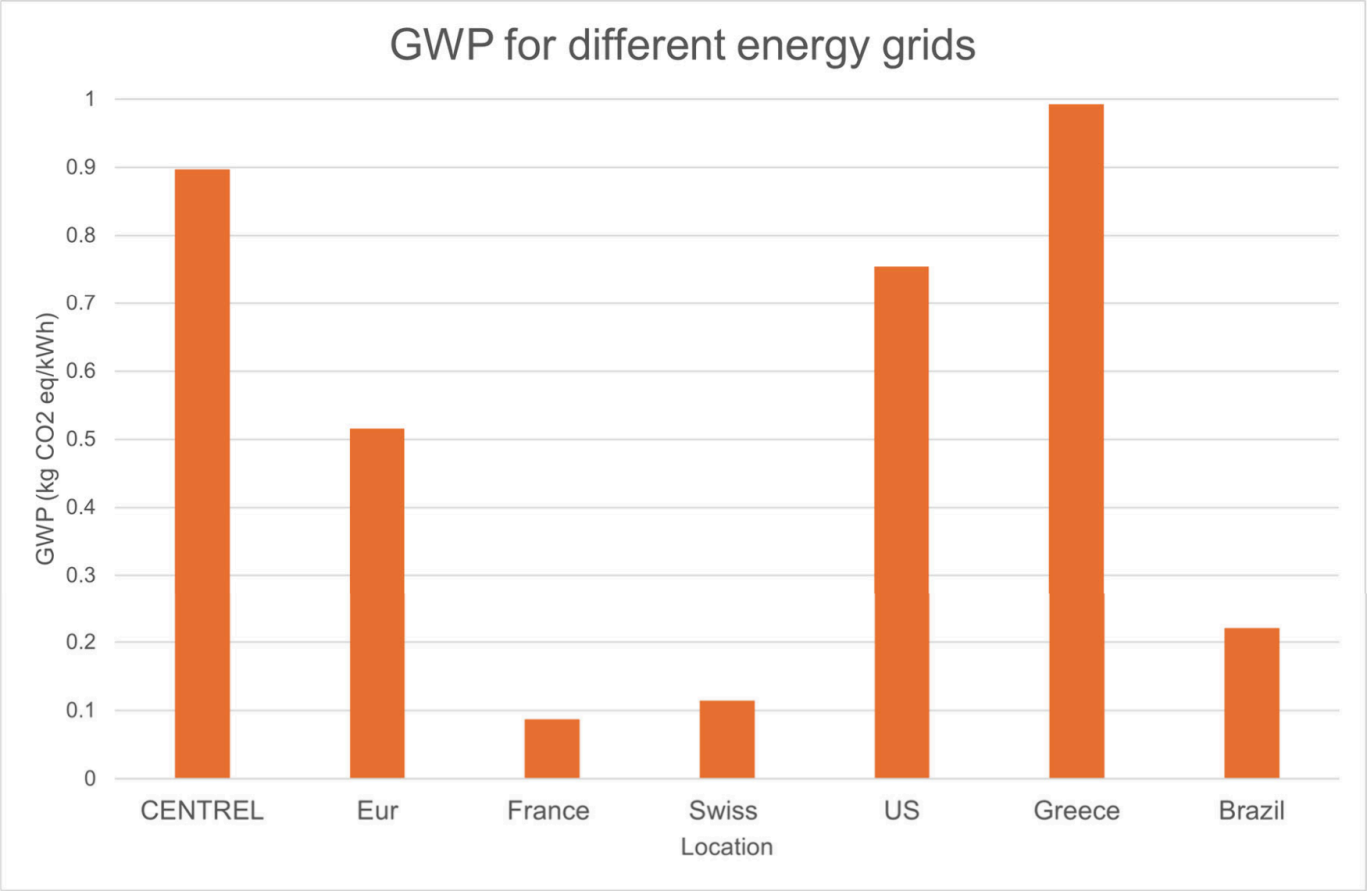
GHG emissions for power production in various production locations. CENTREL (Average Central American), Eur (Average European), France, Switzerland, US (Average US), Greece, and Brazil.

Therefore, if the production country’s energy mix includes a higher proportion of renewable and clean power generation resources, the overall environmental impact will be more favorable for these countries. Figure 5 shows the GWP for different energy grids. PAN fiber production is a highly energy-intensive process, encompassing heated stirring, drawing, stabilization, and carbonization stages. These processes demand substantial energy input, underscoring the importance of utilizing a cleaner energy mix to mitigate the overall GWP associated with PAN fiber production. This can be seen in Figure 4, as the average U.S. production has the greatest CO_2_ emissions, especially for the large energy-dependent processes of fiber annealing and polymer solution. In contrast, France’s cleaner energy grid has a vastly reduced impact on these same energy-dependent processes.

It is crucial to take a holistic view when analyzing manufacturing locations. Infrastructure, transportation, and regulatory standards significantly influence the overall impact of a process.^76^ Relocating production can lead to increased environmental impact due to longer shipping distances for product inputs. Stringent environmental regulations can ensure cleaner production processes.^77^ Additionally, robust infrastructure supports the implementation of efficient manufacturing technologies, potentially further mitigating environmental impacts. Therefore, a comprehensive assessment of these factors is essential in determining the most sustainable and efficient manufacturing locations.

## 4 Conclusion

In this study, we analyzed the environmental impact of novel coaxial PAN/GF fibers produced from recycled wind turbine blade waste. Compared to traditional wind turbine blade disposal methods including landfilling and incineration, our process can produce high-performance CF precursors from landfill-destined waste products. In hopes of dealing with the millions of tons of WTB waste destined. The manufacturing process for PAN precursors was modeled for both US and European markets, with European production showing 5.75% lower emissions than the US counterpart. As production scale increased, total GHG emissions decreased significantly, demonstrating a substantial improvement over lab-scale impacts. Specifically, US production emissions were reduced from 25.68 kg CO_2_ equiv/kg fiber to 3.89 kg CO_2_ equiv/kg fiber, while European production achieved reductions from 19.62 kg CO_2_ equiv/kg fiber to 3.28 kg CO_2_ equiv/kg fiber. To achieve net zero or even negative emissions, it is crucial to explore sustainable raw material inputs. Green chemistry and solvents, along with the use of bio-based or renewable feedstocks, offer promising solutions to further minimize the carbon footprint and toxic emissions associated with manufacturing processes.

This study provides three key insights for researchers, policymakers, and industry stakeholders regarding the environmental impact and feasibility of utilizing wind turbine blade waste for producing value added products with recycled materials.

First, the results demonstrate the potential for upcycling end-of-life wind turbine blades into high-value precursor fibers for carbon fiber production. By quantifying the life cycle environmental benefits, the study highlights how this approach can contribute to the diversion of landfill destined waste and dependence on virgin raw materials. This aligns with broader circular economy goals and supports sustainability efforts in the composites and wind energy industries.

Second, the findings provide a basis for evaluating trade-offs between different processing routes in fiber production. These results can inform decision-making for manufacturers seeking to balance performance, cost, and sustainability in advanced fiber development. It also provides researchers a framework and motivation to analyze their novel techniques to help inform design decisions that can reduce environmental impact.

Third, the study emphasizes the broader implications of integrating recycled materials into carbon fiber supply chains and provides an analysis of a potential solution to the growing WTB waste issue. By demonstrating that wind turbine blade waste can serve as a viable feedstock, this work encourages further exploration of alternative fiber precursors and promotes policy discussions on sustainable end-of-life management strategies for composite materials. These findings may guide future research efforts and industrial adoption of recycled fiber technologies.

## Supplementary Material



## Data Availability

Data requests can be made to Taylor Sobczak via this email: martin.sobczak@uga.edu.

## Notes

Detailed inventory data and calculations used for this life cycle analysis; Table S1 presents the full-scale production inventory data for PAN-GF precursor production, encompassing material and energy inputs across production stages; Table S2 supplies the thermodynamic input values used in solvent recycling, including specific heat and latent heat for methanol and DMF; Equation S1 outlines the formula used to compute the heat energy required to recycle a functional unit of solvent; Table S3 summarizes the calculated environmental impact categories (e.g., global warming potential, ozone depletion, and toxicity metrics) for the solvent recycling process (PDF)

## References

[1] Owusu, P. A. ; Asumadu-Sarkodie, S. . A review of renewable energy sources, sustainability issues and climate change mitigation. In Cogent Engineering; Dubey, S. , Ed.; Taylor & Francis, 2016; Vol. 3, p 1167990. 10.1080/23311916.2016.1167990.

[2] Watson S. (2019). Future emerging technologies
in the wind power sector: A European
perspective. Renewable and Sustainable Energy
Reviews.

[3] Liu P., Meng F., Barlow C. Y. (2022). Wind Turbine
Blade end-of-life options:
An economic comparison. Resources, Conservation
and Recycling.

[4] Liu P., Barlow C. Y. (2017). Wind Turbine
Blade Waste in 2050. Waste Management.

[5] Joustra J., Flipsen B., Balkenende R. (2021). Structural
reuse of high end composite
products: A design case study on wind turbine blades. Resources, Conservation and Recycling.

[6] Fauzi F. N. (2024). Implementation Assessment
of the Offshore Wind Turbine (OWT) for
Remote Regions’ Electrification in Indonesia Based on Geographical
Potential and Economic Attractiveness. Eng.
Sci..

[7] Beauson J. (2022). The complex end-of-life
of wind turbine blades: A review of the European
context. Renewable and Sustainable Energy Reviews.

[8] Ramirez-Tejeda K., Turcotte D. A., Pike S. (2017). Unsustainable
wind turbine blade
disposal practices in the United States: A case for policy intervention
and technological innovation. New Solut..

[9] Osazee I. T. (2021). Landfill
in a sustainable waste disposal. Eur. J. Environ.
Earth Sci..

[10] Deeney P. (2021). End-of-Life alternatives for wind turbine blades: Sustainability
Indices based on the UN sustainable development goals. Resources, Conservation and Recycling.

[11] Bandala E. R. (2021). Emerging materials and
technologies for landfill leachate treatment:
A critical review. Environmental Pollution.

[12] Bobovich B. B. (2019). Glass-fiber
reinforced plastics—construction materials of the sixth technological
paradigm?. Glass Ceramics.

[13] Kabir M. S. (2023). Microplastics in landfill
leachate: Sources, detection, occurrence,
and removal. Environmental science and ecotechnology.

[14] Rathore N., Panwar N. L. (2023). Environmental impact
and waste recycling technologies
for modern wind turbines: An overview. Waste
Management & Research.

[15] Chauhan G. (2018). Novel technologies and conventional processes for recovery
of metals
from waste electrical and electronic equipment: Challenges & opportunities
- A review. Journal of Environmental Chemical
Engineering.

[16] Ball A. S., Stewart R. J., Schliephake K. (2012). A review of the current options for
the treatment and safe disposal of drill cuttings. Waste Management & Research.

[17] Beauson J., Brøndsted P. (2016). Wind turbine
blades: an end of life perspective. MARE-WINT:
New Materials and Reliability in Offshore Wind
Turbine Technology.

[18] Pannucharoenwong N. (2023). The fuel production
for diesel engine from catalytic pyrolysis of
plastic waste. Eng. Sci..

[19] Singh M. V. (2024). Transforming Waste Plastic into High-Value Petrochemical and Diesel
Fraction Through the Pyrolysis Process. ES Energy
Environ..

[20] Yun Y. M. (2014). Pyrolysis characteristics
of GFRP (Glass Fiber Reinforced Plastic)
under non-isothermal conditions. Fuel.

[21] Xu W. (2022). A mini-review of microstructural
control during composite fiber spinning. Polym.
Int..

[22] Chanthee S. (2023). Electrospinning with
natural rubber and Ni doping for carbon dioxide
adsorption and supercapacitor applications. Eng. Sci..

[23] Zander N. E., Gillan M., Sweetser D. (2017). Composite
fibers from recycled plastics
using melt centrifugal spinning. Materials.

[24] Wanassi B́., Azzouz B́., Hassen M. B. (2016). Value-added
waste
cotton yarn: optimization of recycling process and spinning of reclaimed
fibers. Ind. Crops Prod..

[25] Thippanna V. (2024). Coaxial Layered Fiber
Spinning for Wind Turbine Blade Recycling. ACS
Sustainable Chem. Eng..

[26] Frank E., Hermanutz F., Buchmeiser M. R. (2012). Carbon fibers: precursors, manufacturing,
and properties. Macromol. Mater. Eng..

[27] Newcomb B. A. (2016). Processing,
structure, and properties of carbon fibers. Composites Part A: Applied Science and Manufacturing.

[28] Ruland W. (1990). Carbon fibers. Adv. Mater..

[29] Prad̀ere C. (2009). Thermal properties of carbon fibers at very high temperature. Carbon.

[30] Frank E. (2014). Carbon fibers: precursor systems, processing, structure,
and properties. Angew. Chem., Int. Ed..

[31] Kawajiri K., Sakamoto K. (2022). Environmental impact
of carbon fibers fabricated by
an innovative manufacturing process on life cycle greenhouse gas emissions. Sustainable Materials and Technologies.

[32] Sakamoto K. (2022). Impact of the manufacturing
processes of aromatic-polymer-based carbon
fiber on life cycle greenhouse gas emissions. Sustainability.

[33] Geisler G., Hofstetter T. B., Hungerbuhler K. (2004). Production of fine and specialty
chemicals: procedure for the estimation of LCIs. International Journal of Life Cycle Assessment.

[34] Sharma M. M., Mahajani S. M. (2002). Industrial applications
of reactive distillation. Reactive distillation:
Status and future directions.

[35] Shrimali G., Lynes M., Indvik J. (2015). Wind energy deployment in the US:
An empirical analysis of the role of federal and state policies. Renewable and Sustainable Energy Reviews.

[36] Bare, J. ; Tool for the Reduction and Assessment of Chemical and other Environmental Impacts (TRACI); US Environmental Protection Agency: Washington, DC, USA, 2012.

[37] Bare J. (2011). TRACI 2.0:
the tool for the reduction and assessment of chemical and other environmental
impacts 2.0. Clean Technologies and Environmental
Policy.

[38] Bare J. C. (2002). TRACI:
The tool for the reduction and assessment of chemical and other environmental
impacts. J. Ind. Ecol..

[39] Likens G. E. (1979). Acid rain. Sci. Am..

[40] Sundmacher, K. ; Kienle, A. Reactive distillation: status and future directions; John Wiley & Sons, 2006.

[41] Ryberg M. (2014). Updated US and Canadian normalization factors for TRACI
2.1. Clean Technologies and Environmental Policy.

[42] Amin M. (2022). Hydrogen production
through renewable and non-renewable energy processes
and their impact on climate change. Int. J.
Hydrogen Energy.

[43] Khoo H. H., Isoni V., Sharratt P. N. (2018). LCI data
selection criteria for a
multidisciplinary research team: LCA applied to solvents and chemicals. Sustainable Production and Consumption.

[44] Rice K. C., Herman J. S. (2012). Acidification of
Earth: An assessment across mechanisms
and scales. Appl. Geochem..

[45] Hessel V. (2022). Sustainability of green
solvents-review and perspective. Green Chem..

[46] Cooperman A., Eberle A., Lantz E. (2021). Wind turbine
blade material in the
United States: Quantities, costs, and end-of-life options. Resources, Conservation and Recycling.

[47] Diez-Cañamero B., Mendoza J. M. F. (2023). Circular
economy performance and carbon footprint of
wind turbine blade waste management alternatives. Waste Management.

[48] Kelsey N., Meckling J. (2018). Who wins in renewable energy? Evidence
from Europe
and the United States. Energy Research &
Social Science.

[49] Izadian A., Girrens N., Khayyer P. (2013). Renewable energy policies:
A brief
review of the latest US and EU policies. IEEE
Ind. Electron. Mag..

[50] Das S. (2011). Life cycle
assessment of carbon fiber-reinforced polymer composites. International Journal of Life Cycle Assessment.

[51] Maïzi N., Assoumou E. (2014). Future prospects for
nuclear power in France. Appl. Energy.

[52] Larsen K. (2009). Recycling
wind turbine blades. Renew. Energy Focus.

[53] Chen J., Wang J., Ni A. (2019). Recycling
and reuse of composite
materials for wind turbine blades: An overview. J. Reinforced Plastics Compos..

[54] Rybicka J., Tiwari A., Leeke G. A. (2016). Technology readiness
level assessment
of composites recycling technologies”. Journal of Cleaner Production.

[55] Davey S. G. (2018). Sustainability:
Sweet new route to acrylonitrile. Nat. Rev.
Chem..

[56] Nôtre J́.
L. (2011). Biobased
synthesis of acrylonitrile from glutamic acid. Green Chem..

[57] Rani M. (2021). A review on recycling
and reuse methods for carbon fiber/glass fiber
composites waste from wind turbine blades. Composites
part B: engineering.

[58] Nagle A. J. (2020). A Comparative Life Cycle Assessment between landfilling and Co-Processing
of waste from decommissioned Irish wind turbine blades. Journal of Cleaner Production.

[59] Lichtenegger G. (2020). Offshore and onshore wind turbine blade waste
material forecast at
a regional level in Europe until 2050. Waste
management.

[60] Franklin R. (2021). Reinforcing carbonized polyacrylonitrile fibers with nanoscale graphitic
interface-layers. Journal of Materials Science
& Technology.

[61] Xu W. (2021). 3D printing for polymer/particle-based processing:
A review. Composites Part B: Engineering.

[62] Ramanathan A. (2024). Highly loaded carbon
fiber filaments for 3Dprinted composites. J.
Polym. Sci..

[63] Wu X.-F. (2013). Electrospinning
core-shell nanofibers for interfacial toughening
and self-healing of carbon-fiber/epoxy composites. J. Appl. Polym. Sci..

[64] Hermansson F., Janssen M., Svanström M. (2019). Prospective study of lignin-based
and recycled carbon fibers in composites through meta-analysis of
life cycle assessments. Journal of cleaner production.

[65] Arnold U. (2018). Energy-efficient carbon
fiber production with concentrated solar
power: process design and techno-economic analysis. Ind. Eng. Chem. Res..

[66] Carbon Fiber Association . Life Cycle Inventory (LCI) of Carbon Fibers. 2022. Accessed: 2024–07–28. https://www.carbonfiber.gr.jp/english/tech/lci.html.

[67] Chae H. G. (2005). A comparison of reinforcement
efficiency of various types of carbon
nanotubes in polyacrylonitrile fiber. Polymer.

[68] Zhang Y. (2013). Tailoring polyacrylonitrile
interfacial morphological structure by
crystallization in the presence of single-wall carbon nanotubes. ACS Appl. Mater. Interfaces.

[69] Chae H. G. (2007). Stabilization and carbonization of gel spun polyacrylonitrile/single
wall carbon nanotube composite fibers. Polymer.

[70] Ravichandran D. (2021). Multiphase direct ink
writing (MDIW) for multilayered polymer/nanoparticle
composites. Additive Manufacturing.

[71] Xu W. (2021). Hierarchically structured
composite fibers for real nanoscale manipulation
of carbon nanotubes. Adv. Funct. Mater..

[72] Song K. (2013). Structural polymer-based
carbon nanotube composite fibers: Understanding
the processing-structure-performance relationship. Materials.

[73] Ravichandran D. (2024). 3D Printing
Carbon-Carbon Composites With Multilayered Architecture for Enhanced
Multifunctional Properties. J. Mater. Chem.
A.

[74] Zamuda, C. Climate Change and the US Energy Sector: Regional Vulnerabilities and Resilience Solutions; Office of Energy Policy and Systems Analysis, US Department of Energy, 2015.

[75] 92 - Paris la Defense (France) Reseau de Transport d’Electricite RTE . Electrical energy statistics for France. In International Atomic Energy Agency (IAEA), INISFR-10-0381, 2009.

[76] Saxe S., Kasraian D. (2020). Rethinking environmental LCA life stages for transport
infrastructure to facilitate holistic assessment. J. Ind. Ecol..

[77] Bojarski A. D. (2009). Incorporating environmental impacts and regulations in a holistic
supply chains modeling: An LCA approach. Comput.
Chem. Eng..

