# Supplementary material for: Life Cycle Analysis of Coaxial Layered Fiber Spinning for Wind Turbine Blade Recycling
Source: ACS Sustain Resour Manag. 2025 Apr 25;2(5):721–32. doi: 10.1021/acssusresmgt.4c00434 (PMC12105005; doi:10.1021/acssusresmgt.4c00434)
Supplement: Supplementary file 1 [file rm4c00434_si_001.pdf]

Supplementary Materials for

Life Cycle Analysis of Coaxial Layered Fiber Spinning for Wind Turbine Blade Recycling

M. Taylor Sobczak[1], Gengyang Li[2], Arunachalam Ramanathan[1], Sri Vaishnavi Thummalapalli[1], Varunkumar Thippanna[1], Lindsay B. Chambers[1], Taylor Theobald[1], Hongyue Sun[1], Stephen Nolet[3], Ke Li[2,*], Kenan Song[1,*]

[1]Mechanical Engineering, College of Engineering, University of Georgia, Athens, GA, 30605
[2]Environmental Engineering, College of Engineering, University of Georgia, Athens, GA, 30605
[3]TPI Composites, Scottsdale, AZ, 85253

This file includes the following:

- **Table S1**. Full-scale production inventory data for PAN-GF precursor production.
- **Table S2**. Heat energy inventory data for solvent recycling.
- **Equation S1**. Equation used to calculate the heat energy used to recycle a functional unit of solvent in the manufacturing process.
- **Table S3**. Calculated LCA data for the solvent recycling process.

Table S1. Inventory data for full-scale production

Task	*Amount Produced*	*Material Requirements*	*Unit*	*Quantity*
Mechanical Recycling	1000 kg	Glass fiber reinforced polymer from wind turbine blades	kg	1,100
		Transport of wind turbine blades	km	1700
		Electricity	MJ	10.28
Preparation of Polymer Feedstock	1000 kg	PAN polymer	kg	773
		DMF Solvent	kg	16.1
		Electricity	MJ	132.48
Dry Jet Wet Spinning	1000 kg	Methanol	kg	50
		Electricity	MJ	23.3
Fiber Drawing	1000 kg	Silicone Oil	kg	64
		Electricity	MJ	33.88
Fiber Annealing	1000 kg	Electricity	MJ	264.96
		Transport to consumer	km	200
		Off-gas production (HCN, CO, CH4, and hydrocarbons)	kg	70
Solvent Recovery	1000 kg	Heat energy (natural gas)	MJ	1390
Hazardous Waste Disposal for all processes	N/A	Hazardous waste	kg	100

Table S2. Input data for heat energy (Q) calculation to be used in solvent recycling calculation.

Heat Energy		
**cp, methanol**	2.53	kJ/kg K
**dT**	44.7	K
**Methanol latent heat**	1175	kJ/kg
**DMF latent heat**	578	kJ/kg

Equation S1: Equation to calculate heat energy (Q) in MJ for the solvent recycling process.

$$Q = \frac{X_1 \cdot X_2 \cdot X_3}{1000} + \frac{X_4 \cdot X_1}{1000} + \frac{X_5 \cdot X_6}{1000}$$

Where:

- **X_1 = Amount of methanol (kg)**
- **X_2 = Specific heat capacity (Cp) of methanol (kJ/kg K)**
- **X_3= Temperature change (ΔT) (K)**
- **X_4= Latent heat of methanol (kJ/kg)**
- **X_5= Amount of DMF (kg)**
- **X_6= Latent heat of DMF (kJ/kg)**

Table S3: LCA data for recycling 1kg methanol and 0.1 kg of dimethylformamide (DMF)

Impact category	*Heat energy*	*Unit*
OD	1.37E-08	kg CFC-11 eq
GWP	7.51E-02	kg CO2 eq
SF	9.94E-04	kg O3 eq
Acidification	3.37E-03	mol H+ eq
ME	1.50E-05	kg N eq
Carcinogenics	1.29E-10	CTUh
Non-C	3.70E-10	CTUh
RE	7.85E-06	kg PM10 eq
Ecotoxicity	1.99E-03	CTUe
